# Mixed Neuroendocrine Carcinoma and Hepatocellular Carcinoma: A Case Report and Literature Review

**DOI:** 10.3389/fsurg.2021.678853

**Published:** 2021-07-14

**Authors:** Jianwei Lan, Deliang Guo, Xian Qin, Baiyang Chen, Quanyan Liu

**Affiliations:** ^1^Department of Hepatobiliary and Pancreatic Surgery, Research Center of Digestive Diseases, Zhongnan Hospital of Wuhan University, Wuhan, China; ^2^Department of General Surgery, Xiangyang Central Hospital, Affiliated of Hubei University of Arts and Science, Xiangyang, China; ^3^Department of General Surgery, Tianjin Medical University General Hospital, Tianjin, China

**Keywords:** primary hepatic neuroendocrine tumor, neuroendocrine tumor, HCC, case reports, heterogeneous malignancies

## Abstract

**Background:** Neuroendocrine tumors are heterogeneous malignancies that originate from the neuroendocrine system. Previous studies show that this cancer type mainly localizes in the gastrointestinal tract and often metastasizes to the liver. Primary liver neuroendocrine tumors are very rare and primary hepatic neuroendocrine tumors (PHNET) with concurrent hepatocellular carcinoma (HCC) are extremely rare. To the best of our knowledge, only few PHNET cases have been identified, making their diagnosis difficult. Here, we report the biggest ever reported and “deceiving” lesion of a mixed neuroendocrine-non-neuroendocrine neoplasm in the liver, aiming to raise awareness and improve treatment of the disease.

**Case Presentation:** Here, we report a preoperative misdiagnosed case that presented with hepatocellular carcinoma clinical features and no extrahepatic tumors. Postoperative pathology confirmed that it was a mixed neuroendocrine-non-neuroendocrine neoplasm. The patient was then referred for etoposide and cisplatin-based chemotherapy. No disease recurrence was observed at the 6-month follow-up.

**Conclusion:** We report a very rare and easily misdiagnosed case and we speculate that there were “undifferentiated cells” undergoing neuroendocrine and hepatocellular carcinoma differentiation, during which some hepatocellular carcinoma cells express neuroendocrine features. We recommend proper surgery and postoperative platinum-based chemotherapy in the management of this disease.

## Introduction

Neuroendocrine tumors are heterogeneous malignancies that originate from the neuroendocrine system. Previous studies show that this cancer type mainly localizes in the gastrointestinal tract, including the small intestine (30.8%), rectum (26.3%), colon (17.6%), pancreas (12.1%), and appendix (5.7%), and often metastasizes to the liver (1). Primary liver neuroendocrine tumors are very rare and primary hepatic neuroendocrine tumors (PHNET) with concurrent hepatocellular carcinoma (HCC) are extremely rare. To the best of our knowledge, only few PHNET cases have been identified. It is rarer the two components occur simultaneously, making their diagnosis difficult. Here, we report the biggest ever reported lesion of a mixed neuroendocrine-non-neuroendocrine neoplasm confirmed by postoperative pathology in the liver, aiming to raise awareness and improve treatment of the disease.

## Case Presentation

**Figure 1:**
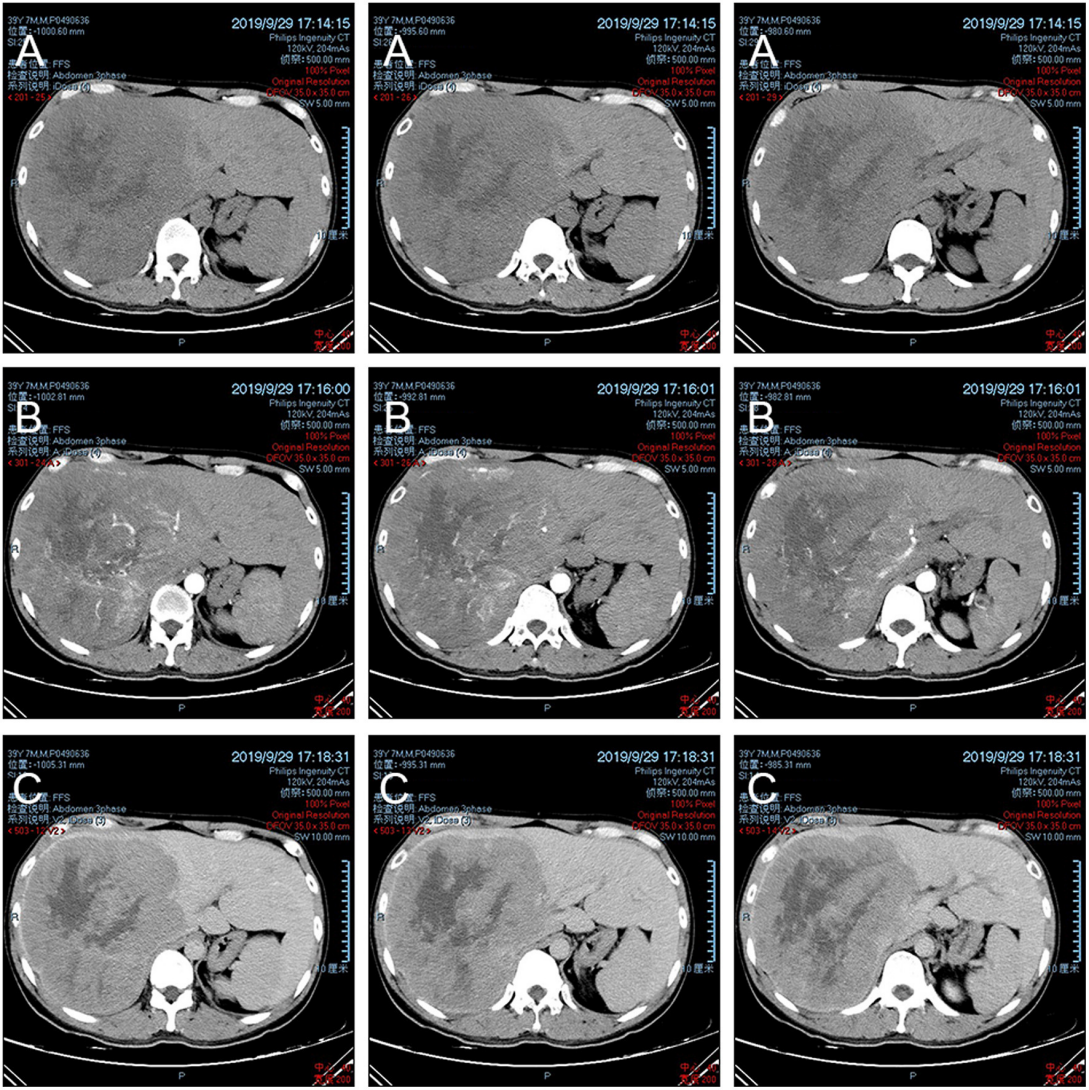
CT images. **(A)** Plain scan; **(B)** arterial phase; and **(C)** portal venous phase.

A 39-year-old man without known, significant medical history, was admitted to our department with >2 months of anorexia. The patient mainly complained of the discovery of focal liver lesions for 7 days. He denied any tobacco or alcohol use and any treatments. His family history did not reveal liver disease. At admission, the patient was afebrile and had normal vital signs. Physical examination yielded normal findings. Laboratory tests revealed: total bilirubin = 23.7 μmol/L↑ (reference range: 5–21 μmol/L), direct bilirubin = 5.6 μmol/L (reference range: 0–7 μmol/L), indirect bilirubin = 18.1 μmol/L↑ (reference range: 1.5–18 μmol/L), γ-glutamyl transpeptidase = 125U/L↑ (reference range: 8–57 U/L), AFP serum levels = 22468.30 ng/mL↑ (reference range: 0–6.6 ng/mL), and normal CA19-9 (carbohydrate antigen 19–9) and CA125 (carbohydrate antigen 125) serum levels. The patient was HBsAg (hepatitis B surface antigen) and HBc-Ab (hepatitis B core antibody) positive and had a hepatitis B DNA copy number of 1.03E3IU/mL↑. Abdominal computed tomography (CT) (Figure 1) revealed marked liver enlargement and a large soft tissue mass on the right hepatic lobe with an estimated volume of 17.4 × 16.1 × 20.1 cm. The mass exhibited uneven internal density with multiple dotted high-density and flaky low-density shadows. The lesion was enhanced in the early phase and washed out in the delayed phase. Display of the right branch of portal vein was unclear. Given these results, hepatic cancer with portal vein cancerous thrombus was suspected.

**Figure 2:**
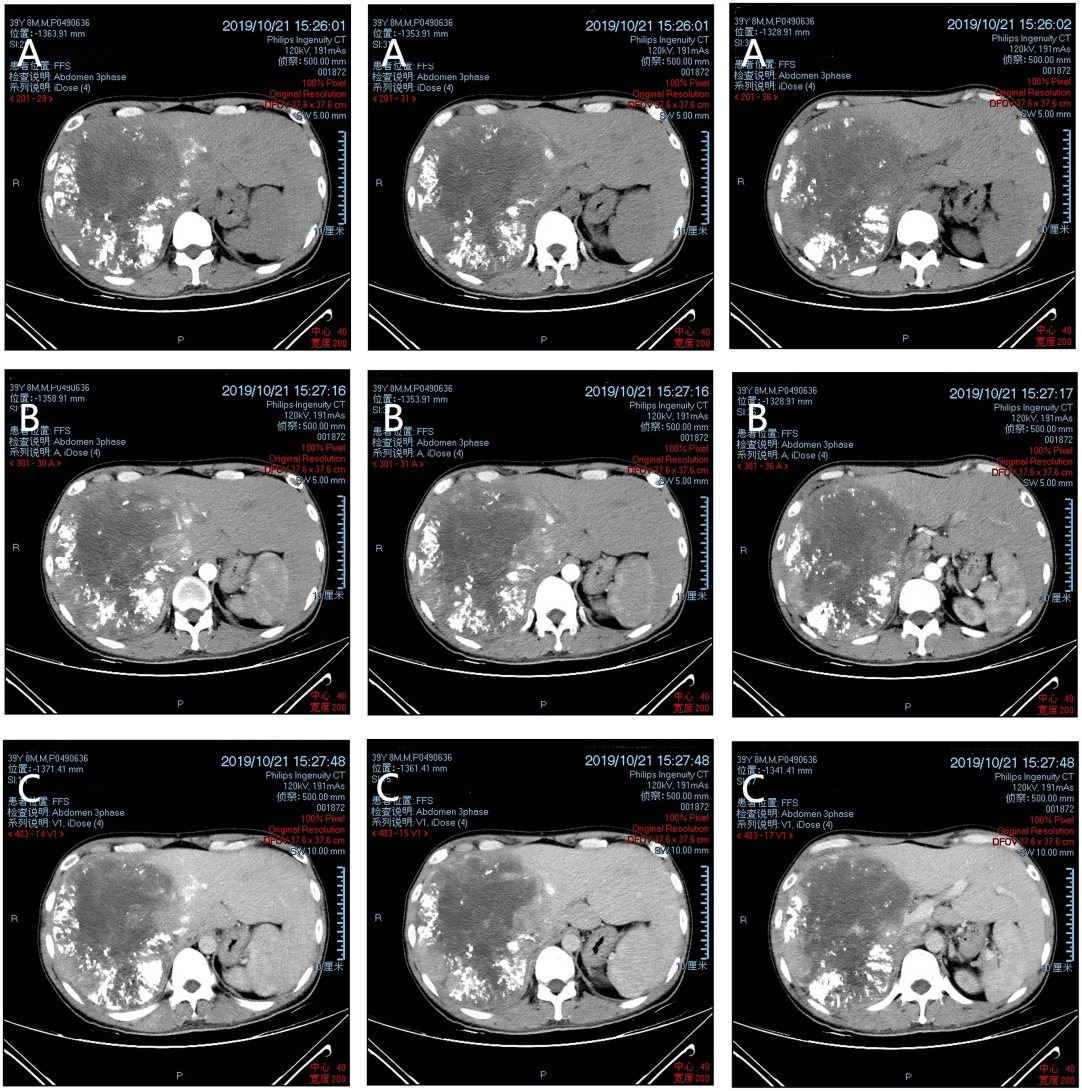
CT images after TACE. **(A)** Plain scan; **(B)** arterial phase; and **(C)** portal venous phase.

On the 3rd day after admission, TACE (transcatheter arterial chemoembolization) was used to embolize the tumor feeding arteries to slow tumor development. An emulsion of oxaliplatin (50 mg) and lipiodol emulsion (20 mL) was administered via the feeding arteries and embolization performed using a gelatin sponge. The patient was then put on home-based recuperation. After 2 weeks, serum bilirubin was observed to have returned to normal levels, while AFP levels fell to 458.20 ng/mL relative to 22468.30 ng/mL at admission (reference range: 0–6.6 ng/mL). Figure 2 was a reexamination of CT 2 weeks after TACE, in which tumor growth was not observed and a large portion of the tumor was necrotic, and good results were achieved. Then an extended right hemihepatectomy laparotomy was performed by laparotomy under general anesthesia. During surgical exploration, laparotomy revealed the mass's bumpy surface. The remaining liver tissue was normal without obvious pathological changes. The operation did not reveal asities or liver cirrhosis. Extensive abdominal exploration did not find additional primary tumor sites.

**Figure 3:**
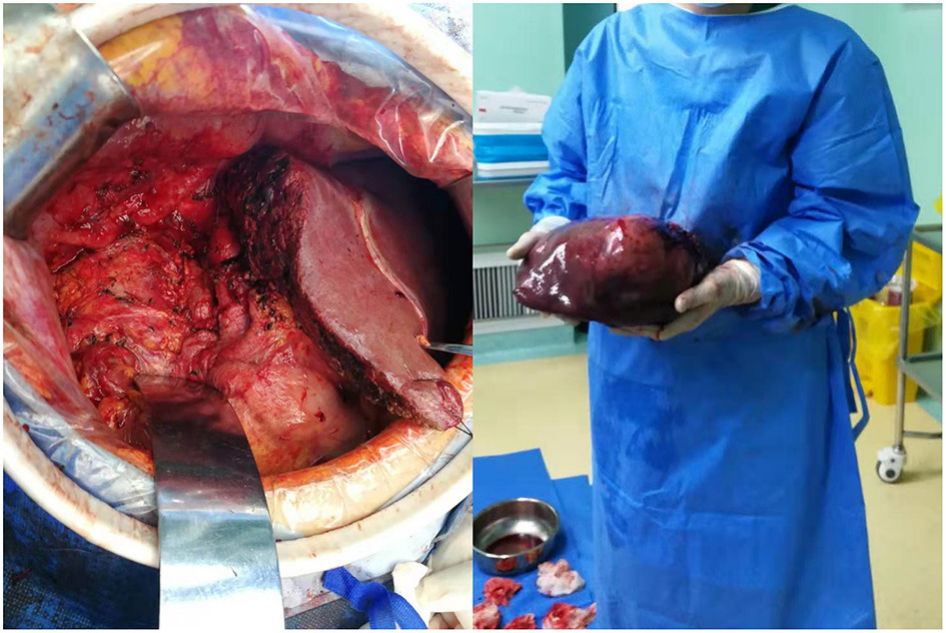
Tumor appearance. (**Left)** Operating field after excision of right, caudate and quadrate lobe of liver; (**Right**) gross specimen.

**Figure 4:**
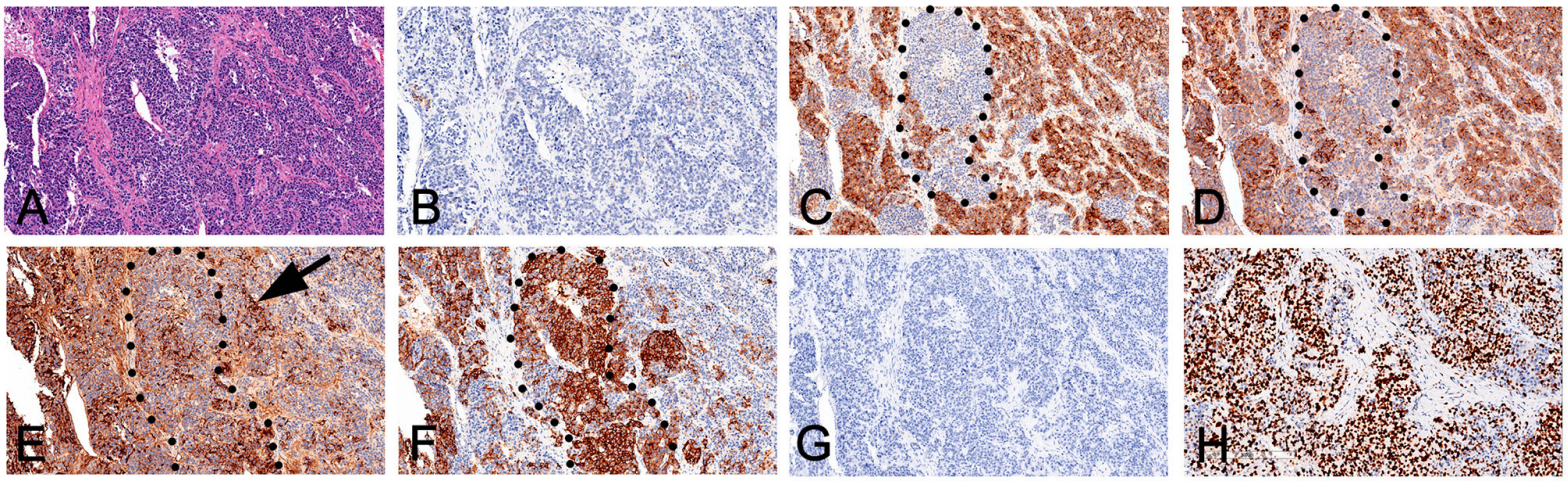
Immunohistochemical expression. **(A)** HE staining; **(B)** Hepatocyte(+); **(C)** Glypican-3 (+); **(D)** AFP (+); **(E)** CgA (+); **(F)** SYN (+); **(G)** Arginase-1 (–); and **(H)** KI-67.

The resected liver tissue had a volume of 26 × 23 × 12 cm (Figure 3). Multi-section incision revealed a 21 × 20 × 12 cm mass that was gray-yellow/gray-brown, and soft to touch, with geographic necrosis. The rest of the liver tissue section was grayish red. Microscopically, the neoplastic cells were disposed in nets and sheets. The tumor is mainly comprised of neoplastic cells with enlarged nuclei, inconspicuous nucleoli, and granular chromatin, arranged in nests or rosette structures. Nuclear molding change and mitoses were frequently observed (Figure 4A). The resected liver tissue had a volume of 26 × 23 × 12 cm (Figure 3). Multi-section incision revealed a 21 × 20 × 12 cm mass that was gray-yellow/gray-brown, and soft to touch, with geographic necrosis. The rest of the liver tissue section was grayish red. Microscopically, the neoplastic cells were disposed in nets and sheets. The tumor is mainly comprised of neoplastic cells with enlarged nuclei, inconspicuous nucleoli, and granular chromatin, arranged in nests or rosette structures. Nuclear molding change and mitoses were frequently observed (Figure 4A). A small nest strongly expressed SYN on the cell membrane (Figure 4F). The small round cells on the right side of the dotted line lost this staining pattern, where the cells on the left side of the dotted line still remained this pattern. The cells outside the dotted line expressed GPC and AFP diffusely (Figures 4C,D). And the small nest showed weak AFP expression. All small round cells showed CgA positivity (Figure 4E). The Ki-67 ratio was about 70%. Postoperative pathological findings (Figure 4) confirmed a mixed neuroendocrine-non-neuroendocrine neoplasm. Postoperative pathological findings (Figure 4) confirmed a mixed neuroendocrine-non-neuroendocrine neoplasm. The patient was referred to an internist for etoposide and cisplatin-based chemotherapy. At 6-month follow-up, no recurrence was seen and the patient had remained disease-free.

## Discussion and Conclusion

Here, we report a very rare and easily misdiagnosed case. To the best of our knowledge, this is so far, the largest recorded mixed neuroendocrine-non-neuroendocrine neoplasm. Multiple points deserve close attention. First, clinical examination was highly suggestive of hepatocellular carcinoma (HCC) (1). The lesion was enhanced in the early phase and washed out in the delayed phase with possible simultaneous portal vein invasion (2). The patient was infected with HBV and had abnormal AFP levels. However, postoperative pathology revealed a mixed neuroendocrine-non-neuroendocrine neoplasm, highlighting the importance of pathological diagnosis. Second, some cells were positive for neuroendocrine carcinoma (NEC) and HCC markers, indicating that some “undifferentiated cells” were plastic during the differentiation process, irrespective of whether they were hepatic malignant tumor cells or hepatic progenitor cells.

Primary neuroendocrine tumors with HCC in the liver are extremely rare (2). The first case of HCC with carcinoid tumors was reported in 1984 (3). The lesions fall into 2 classes, collision and combined (4). Collision type tumors are distinguished by fibrillar component, while in combined type tumors the 2 features are mixed and cannot be recognized. Microscopically, they fall into 3 types, transitional, intermediate, and separate. The former 2 types represent colocalization of neuroendocrine components and non-neuroendocrine components. In the transitional type, NEC and HCC components intermingle in transitional areas. In the intermediate type, intermediate components simultaneously express hepatocyte markers and neuroendocrine markers, intermingling with the NEC and HCC components. In the separate type, the 2 features occur independently (5). Neuroendocrine tumors are known to mainly localize in the gastrointestinal tract, including the small intestine (30.8%), rectum (26.3%), colon (17.6%), pancreas (12.1%), and appendix (5.7%), and to most frequently metastasize to the liver (1). Primary neuroendocrine tumors in the liver are very rare. Information about primary mixed NEC and HCC is summarized in Table 1 (2–4, 6–18). Most primary neuroendocrine tumor patients have underlying liver disease. The presence of hepatitis B and C virus suggests a chronic process. Most patients are middle-aged and elderly men and the majority have the combined type. Tumor markers are usually manifested with primary liver cancer's characteristics, evaluated AFP and normal CA125/199 levels.

**Table 1:** Summary of reported cases.

**Author**	**Year**	**Age**	**Sex**	**Symptoms**	**Hepatitis virus**	**Tumor marker**	**Location**	**Portal vein invasion**	**Size of tumor**	**Solitary or multiple**	**Background of the liver**	**Synchronous metastases**	**Type**	**Ki-67**	**Treatment**	**Chemotherapy protocol**	**Recurrence site & Time**	**Clinical course/death causes**
Barsky	1983	43	Man	Right upper quadrant swelling	HBV	AFP↑	Right lobe	NM	Large	Solitary	Cirrhotic	Omentum	Combined	NM	Chemotherapy	Adriamycin,5-fluorouracil	NM	26 months death/ liver insufficiency
Artopoulos	1994	69	Man	Mild abdominal pain	HBV	AFP↑	NM	NM	100 mm	Solitary	Cirrhotic	NM	Combined	NM	Operation	None	NM	NM
Vora	2000	63	Man	Abdominal pain and jaundice	NM	NM	NM	NM	100 mm	Solitary	Cirrhotic	NM	Combined	NM	Operation	None	NM	Death/ Perioperative complication
Tajima	1992	73	Female	General malaise/ nausea/ abdominal pain	None	NM	Right lobe	NM	50 mm	Solitary	Non-cirrhotic	NM	Combined	NM	NM	None	None	7 days death/ disease progression
Ishida	2003	72	Man	None	HCV	AFP↑	Segment 8/5	NM	30 and 15 mm	Multiple	Cirrhotic	None	Collision	High	Operation	None	NM	NM
Yamaguchi	2004	71	Man	None	HCV	AFP↑, CEA N	Segment 5/6	NM	41*40 mm/45 *40 mm	Multiple	Fibrosis	NM	Combined+ collision	51.1 ± 13.1%	Operation	None	Pelvic (5 months)	5 months alive
Garcia	2006	50	Man	None	HCV	AFP↑, CEA/ CA125↑	IVb/V	NM	53*45 *40 mm	Solitary	Non-cirrhotic	None	Collision	70–80%	Operation → TACE → chemotherapy	Cisplatin → doxorubicin → thalidomide and bevacizumab	Right liver's posterior and anterior segments (4 months)	16 months alive
Yang	2009	65	Man	intermittent epigastric pain	HBV	AFP/ CEA/ CA199 N	Right lobe	NM	75 mm	Solitary	Non-cirrhotic	Regional lymph node	Combined	Higher	Operation	None	Liver and bilateral adrenal glands and paraaortic lymphnodes (3 months)	12 months death/disease progression
Nakanishi	2012	76	Man	None	HCV	AFP↑, PIVKA-II/ CEA/ CA199 N	Segment 6	NM	30 and 15 mm	Solitary	Non-cirrhotic	None	Combined	NM	TACE → operation	None	Sacral bone (6 months)	7 months death/disease progression, aspiration pneumonia
Aboelenen	2013	51	Man	Dull aching abdominal pain	HCV	AFP↑, CEA/ CA125↑	Right hemiliver	No	75 mm	Solitary	Non-cirrhotic	None	Combined	NM	Operation	None	None	6 months alive
Baker	2016	76	Man	None	None	AFP↑	Left liver	Yes	55 mm	Solitary	Cirrhotic	None	Collision	50%	Operation	None	None	NM
Choi	2016	72	Man	None	HCV	AFP N, PIVKA-II↑	Segment 3	NM	25* 20 mm	Solitary	NM	None	Collision	NM	Operation+ chemotherapy	Etoposide, cisplatin	Right hepatic lobe (6 months)	10 months alive
Nishino	2016	72	Man	None	HCV	AFP /PIVKA-II↑, CA199/ 125 N	Segment 8/6	No	20 mm/ 10 mm	Multiple	Cirrhotic	None	Combined	80%	Operation → chemotherapy	Cisplatin and etoposide	Regional and paraaortic lymphaden (1 week)	2 months death/disease progression
Nomura	2016	71	Man	NM	HCV	AFP↑	Segment 5	NM	41*40 mm	Solitary	Non-cirrhotic	NM	Combined	Higher	Operation	None	Intrahepatic metastasis	8.6 months death/ liver failure
Nomura	2016	71	Man	NM	HCV	AFP↑	Segment 5/8	NM	30* 10 mm	Multiple	Non-cirrhotic	NM	Collision	Higher	RFA → Operation	None	Intrahepatic metastasis	2.6 months death/ liver failure
Nomura	2016	50	Man	NM	HBV	AFP↑	Segment 3	NM	18* 17 mm	Solitary	Cirrhotic	NM	Combined	Higher	Operation	None	Intrahepatic metastasis	19.5 months alive
Nomura	2016	63	Man	NM	HCV	AFP↑	Segment 8	NM	30* 30 mm	Solitary	Non-cirrhotic	NM	Combined	Higher	IFN → Operation	None	Intrahepatic metastasis	24 months alive
Yun	2016	68	Female	None	HBV	AFP↑, CEA/ CA199/ PIVKA-II N		None	24 mm	Solitary	Cirrhotic	NM	Combined	NM	Operation → chemotherapy/ radiation	Cisplatin	Right scapula bone (6 months)	9 months death/ disease progression
Lu	2017	65	Man	Right upper quadrant pain	None	AFP↑	Right lobe	Yes	140* 140* 80 mm	Solitary	Non-cirrhotic	Gallbladder	Combined		Hospice care	None	Colon (1 months)	1 months alive
Okumura	2017	70	Man	None	HCV	AFP/ CEA/ CA199 N	Segment 7/8	NM	110* 100 mm	Solitary	Non-cirrhotic	None	Combined+ collision	3–20%	TACE+ PTPE → operation → chemotherapy+ radiation therapy	sorafenib	lymph nodes/ lumbar vertebras (1 month)	3 months death/NM
Liu	2017	65	Man	Abdominal discomfort	HCV	AFP↑	Segment 4	No	43* 29* 24 mm		Cirrhotic	Regional lymph node	Collision	>80%	Operation	None	NM	1.3 months death/ deteriorating liver and renal functions
Kwon	2018	44	Man	None	HBV	PTH/ NSE↑	Segment 8/6	Yes	105* 80 mm/13* 10 mm	Multiple	Cirrhotic	None	Collision	NM	Operation → chemotherapy/ radiation	One cycle of 5-flourouracil chemotherapy	Liver and whole skeleton (59 days)	2.3 months death/ disease progression
Matsumoto	2017	77	Man	NM	NM	NM	Segment 4/ 6/ 8	NM	40 mm	Solitary	NM	None	Combined	NM	Operation	None	Liver (3 months)	3 months death/disease progression
Yilmaz	2018	56	Man	Abdominal distension related to ascites	None	AFP/ CEA/ CA199 N		NM	23 mm	Multiple	Cirrhotic	None	Collision	NM	Liver transplantation	None	None	10 months alive

For our patient, immunohistochemical analysis revealed the NEC component to be positive for both CgA and SYN, and negative for Glypain-3, which is usually positive in HCC. Interestingly, some HCC components were positive for neuroendocrine markers (Figure 4E, Arrow). Moreover, NEC cells were poorly positive for AFP.

Where do these NEC cells come from? Gould et al., have suggested that they may originate from neuroendocrine differentiation of a single malignant stem cell or a precursor of other hepatic malignant tumors (19). Pettinato et al. hypothesized that hepatic progenitor cells translocate to the intrahepatic bile duct epithelium during embryonic development and may have the capacity to progress into NECs (6). Both hepatic malignant tumors or hepatic progenitor cells are capable of differentiating toward a hepatocellular or biliary fate. Thus, we speculate that benign or malignant undifferentiated cells may have undergone hepatocellular and neuroendocrine differentiation during proliferation, with the 2 components intermingling. As Figure 4 showed, hepatocellular dominant areas were strongly positive for hepatocyte-related antibodies and poorly positive for neuroendocrine markers. In neuroendocrine dominant areas, the reverse was observed. While intermediate cells stained positive for both hepatocyte and neuroendocrine markers. Due to their merging, it was impossible to clearly define the cancer type. Based on 2019 WHO classification of digestive system tumors (20), we diagnosed the case as a mixed neuroendocrine-non-neuroendocrine neoplasm (MiNEN). Thus, some PHNET with AFP or other index changes may not be real PHNET, but hepatic progenitor cells undergoing hepatocellular neuroendocrine differentiation, and may fall under the MiNEN category.

Surgical resection is the first choice of treatment in cases of pure PHNET (21, 22). Studies by Givi et al. have shown that the median survival time of patients undergoing surgery is about 159 months, while that of patients without surgical treatment is only 47 months (23). However, the prognosis of HCC with PHNET remains unclear due to its rarity. A significantly higher Ki-67 proliferative index in NEC relative to HCC suggests a poorer prognosis (5). In PHNET, laboratory tests reveal some tumor markers, including AFP, CEA, and CA-199 to often be within the normal range. Indicators of hepatitis, cirrhosis, and other hepatic diseases are also negative. However, these indicators change accordingly when PHNET combines with HCC. Final diagnosis still requires histopathological analysis and careful exclusion of extrahepatic primary tumor.

In conclusion, we reported a rare preoperative misdiagnosis case showing deceiving clinical features. Postoperative pathology confirmed the final diagnosis. We speculate that there were “incomplete differentiation cells” undergoing neuroendocrine and hepatocellular carcinoma differentiation because of HBV infection or other chronic hepatic diseases, which could explain some hepatocellular carcinoma cells could express neuroendocrine features simultaneously. We recommend proper surgery and postoperative platinum-based chemotherapy in the management of this disease. We will also conduct a long-term follow-up of the patient in this article to better understand the disease.

## Data Availability Statement

The original contributions presented in the study are included in the article/supplementary material, further inquiries can be directed to the corresponding author/s.

## Ethics Statement

This case report was approved in full by the Ethics Committee of the Zhongnan Hospital of Wuhan University (Wuhan, China). Written informed consent was obtained from this patient. Data were collected from the daily medical nursing records by staff experienced in gathering clinical information.

## Author Contributions

JL and QL designed the idea. XQ and BC collected the data. JL and DG processed the data and wrote the manuscript. All authors have read and approved the manuscript.

## Conflict of Interest

The authors declare that the research was conducted in the absence of any commercial or financial relationships that could be construed as a potential conflict of interest.

## Abbreviations

PHNET: Primary hepatic neuroendocrine tumor
AFP: alpha-fetoprotein
CEA: carcinoembryonic antigen
CA199: carbohydrate antigen 199
CA125: carbohydrate antigen 125
NEC: neuroendocrine carcinoma
HCC: Hepatocellular carcinoma
SCNEC: small cell neuroendocrine carcinoma
LCNEC: large cell neuroendocrine carcinoma
MiNEN: mixed neuroendocrine–non-neuroendocrine neoplasm.
